# Supplementary material for: Semi-field life-table studies of Aedes albopictus (Diptera: Culicidae) in Guangzhou, China
Source: PLoS One. 2020 Mar 18;15(3):e0229829. doi: 10.1371/journal.pone.0229829 (PMC7080243; doi:10.1371/journal.pone.0229829)
Supplement: S1 Table — (DOC) [file pone.0229829.s004.doc]

| Experiment | Survival rate | | |
| --- | --- | --- | --- |
|  | Egg hatch | Pupation | Emergence |
| June | 56.1 [43.4, 68.8] AB | 92.8 [78.4, 100] A | 87.8 [74.1, 100] A |
| July | 35.5 [24.2, 46.8] B | 92.2 [87.7, 96.6] A | 87.9 [80.9, 94.9] A |
| August | 67.8 [30.3, 100] A | 90.1 [83.3, 97.0] A | 83.3 [69.2, 97.4] A |
| September | 69.8 [52.3, 87.2] A | 93.4 [86.7, 100] A | 80.2 [68.6, 91.8] A |
| October | 77.1 [74.8, 79.4] A | 83.4 [79.8, 87.1] A | 79.2 [70.4, 88.0] A |
| November | 54.1 [26.9, 81.4] AB | 81.6 [65.6, 97.6] A | 76.8 [60.5, 93.1] A |
|  | Cumulative development time | | |
|  | Egg hatch | 1st-2nd instar | 3rd-4th instar |
| June | 7.3 [6.4, 8.1] A | 8.2 [7.4, 9.0] A | 10.8 [10.3, 11.4] A |
| July | 2.1 [1.6, 2.6] C | 2.8 [2.5, 3.0] B | 5.5 [ 5.1, 5.8] B |
| August | 3.2 [2.4, 4.0] BC | 3.4 [2.7, 4.2] B | 5.8 [ 5.1, 6.4] B |
| September | 2.4 [1.6, 3.3] C | 3.0 [2.0, 4.0] B | 5.1 [ 4.1, 6.0] B |
| October | 2.4 [1.2, 3.5] C | 3.7 [3.6, 3.8] B | 6.4 [ 6.0, 6.9] B |
| November | 4.7 [1.4, 8.0] B | 7.4 [2.0, 12.8] A | 14.4 [ 7.9, 20.9] A |
|  | Cumulative development time (con't) | | |
|  | Pupa | Female | Male |
| June | 13.2 [12.7, 13.6] AB | 15.1 [14.3, 16.0] B | 15.1 [14.7, 15.5] B |
| July | 7.6 [ 7.2, 8.0] C | 9.6 [ 9.1, 10.1] C | 9.2 [ 8.8, 9.6] C |
| August | 7.4 [ 6.7, 8.1] C | 9.1 [ 8.3, 9.9] C | 9.6 [ 8.9, 10.2] C |
| September | 6.9 [ 6.0, 7.8] C | 8.9 [ 7.8, 10.0] C | 8.5 [ 8.1, 8.9] C |
| October | 9.1 [ 8.6, 9.5] BC | 11.6 [11.3, 11.9] C | 11.5 [10.5, 12.5] BC |
| November | 17.4 [ 9.5, 25.2] A | 24.1 [18.2, 30.0] A | 21.5 [12.6, 30.5] A |

** Levels not connected by the same letter are significantly different. Significance was determined using the Tukey HSD test at 0.05 level.
