# Supplementary material for: Semi-field life-table studies of Aedes albopictus (Diptera: Culicidae) in Guangzhou, China
Source: PLoS One. 2020 Mar 18;15(3):e0229829. doi: 10.1371/journal.pone.0229829 (PMC7080243; doi:10.1371/journal.pone.0229829)
Supplement: S2 Table — (DOCX) [file pone.0229829.s005.docx]

S2 Table. Adult life-table summary

| Experiment | Survival time | Daily survival rate | Life-time egg mass |
| --- | --- | --- | --- |
| June | 3.8 [2.4, 5.3] B | 85.8 [82.4, 89.2] A | 67.6 [49.0, 86.2] A |
| July | 4.3 [3.6, 5.0] B | 82.2 [80.9, 83.6] A | 70.0 [30.6, 100] A |
| September | 3.8 [2.5, 5.2] B | 81.3 [75.7, 86.9] A | 18.8 [ 7.9, 29.6] B |
| October | 7.0 [4.2, 9.9] A | 87.0 [82.6, 91.5] A | 20.5 [ 0, 49.8] B |
| November | 5.8 [3.4, 8.2] AB | 86.7 [78.9, 94.5] A | 12.4 [ 0, 33.5] B |
