# Supplementary material for: Semi-field life-table studies of Aedes albopictus (Diptera: Culicidae) in Guangzhou, China
Source: PLoS One. 2020 Mar 18;15(3):e0229829. doi: 10.1371/journal.pone.0229829 (PMC7080243; doi:10.1371/journal.pone.0229829)
Supplement: S3 Table — (DOCX) [file pone.0229829.s006.docx]

Supplement Table S3

Table S3. Results of multiple regression and stepwise regression analyses for finding the key factors affecting larval and pupa development and adult survivorship

1. **Egg hatch rate**

| Regression | |  | |  | |  | |  | |  |
| --- | --- | --- | --- | --- | --- | --- | --- | --- | --- | --- |
|  | ANOVA | | R^2^ | | R^2^ adj | | F4,23 | | P | |
|  |  | | 0.55 | | 0.47 | | 7.01 | | 0.0008 | |
|  | Parameter Estimates | | | | | |  | |  | |
|  | Term | | Estimate | | Std Error | | t Ratio | | Prob>\|t\| | |
|  | Intercept | | 252.1547 | | 44.8963 | | 5.62 | | <.0001 | |
|  | Light | | -0.0276 | | 0.0067 | | -4.09 | | 0.0005 | |
|  | Photoperiod | | -13.6901 | | 3.0053 | | -4.56 | | 0.0001 | |
|  | Temperature | | 1.2634 | | 0.8046 | | 1.57 | | 0.1300 | |
|  | Humidity | | -0.2901 | | 0.2190 | | -1.32 | | 0.1982 | |
| Stepwise regression | | | |  | |  | |  | |  |
|  | ANOVA | | R^2^ | | R^2^ adj | | F2,25 | | P | |
|  |  | | 0.4296 | | 0.3840 | | 9.41 | | 0.0009 | |
|  | Parameter Estimates | | | | | |  | |  | |
|  | Term | | Estimate | | Std Error | | t Ratio | | Prob>\|t\| | |
|  | Intercept | | 210.0448 | | 36.4982 | | 5.75 | | <.0001 | |
|  | Light | | -0.0262 | | 0.0071 | | -3.69 | | 0.0011 | |
|  | Photoperiod | | -9.3081 | | 2.5609 | | -3.63 | | 0.0013 | |

1. **Pupation rate**

| Regression | |  | |  | |  | |  | |  |
| --- | --- | --- | --- | --- | --- | --- | --- | --- | --- | --- |
|  | ANOVA | | R^2^ | | R^2^ adj | | F4,23 | | P | |
|  |  | | 0.38 | | 0.27 | | 3.46 | | 0.0237 | |
|  | Parameter Estimates | | | | | |  | |  | |
|  | Term | | Estimate | | Std Error | | t Ratio | | Prob>\|t\| | |
|  | Intercept | | 58.6989 | | 19.9301 | | 2.95 | | 0.0073 | |
|  | Light | | -0.0019 | | 0.0030 | | -0.62 | | 0.5396 | |
|  | Photoperiod | | 1.3328 | | 1.3341 | | 1.00 | | 0.3282 | |
|  | Temperature | | 0.6010 | | 0.3572 | | 1.68 | | 0.1060 | |
|  | Humidity | | -0.0002 | | 0.0972 | | 0.00 | | 0.9984 | |
| Stepwise regression | | | |  | |  | |  | |  |
|  | ANOVA | | R^2^ | | R^2^ adj | | F1,26 | | P | |
|  |  | | 0.3199 | | 0.2937 | | 12.23 | | 0.0017 | |
|  | Parameter Estimates | | | | | |  | |  | |
|  | Term | | Estimate | | Std Error | | t Ratio | | Prob>\|t\| | |
|  | Intercept | | 65.5644 | | 6.9025 | | 9.50 | | <.0001 | |
|  | Temperature | | 0.8901 | | 0.2545 | | 3.50 | | 0.0017 | |

1. **Emergence rate**

| Regression | |  |  |  |  |
| --- | --- | --- | --- | --- | --- |
|  | ANOVA | R2 | R2 adj | F4,19 | P |
|  |  | 0.27 | 0.15 | 2.16 | 0.1062 |
|  | Parameter Estimates | | |  |  |
|  | Term | Estimate | Std Error | t Ratio | Prob>\|t\| |
|  | Intercept | 50.4221 | 24.3089 | 2.07 | 0.0494 |
|  | Light | -0.0022 | 0.0037 | -0.59 | 0.5619 |
|  | Photoperiod | 3.0499 | 1.6272 | 1.87 | 0.0736 |
|  | Temperature | -0.0583 | 0.4357 | -0.13 | 0.8947 |
|  | Humidity | -0.0260 | 0.1186 | -0.22 | 0.8283 |
| Stepwise regression | | |  |  |  |
|  | ANOVA | R2 | R2 adj | F1,26 | P |
|  |  | 0.2615 | 0.2330 | 9.20 | 0.0054 |
|  | Parameter Estimates | | |  |  |
|  | Term | Estimate | Std Error | t Ratio | Prob>\|t\| |
|  | Intercept | 40.9484 | 13.9260 | 2.94 | 0.0068 |
|  | Photoperiod | 3.3461 | 1.1029 | 3.03 | 0.0054 |

1. **Egg hatch time**

| Regression | |  | |  | |  | |  | |  |
| --- | --- | --- | --- | --- | --- | --- | --- | --- | --- | --- |
|  | ANOVA | | R^2^ | | R^2^ adj | | F4,23 | | P | |
|  |  | | 0.71 | | 0.66 | | 13.95 | | <.0001 | |
|  | Parameter Estimates | | | | | |  | |  | |
|  | Term | | Estimate | | Std Error | | t Ratio | | Prob>\|t\| | |
|  | Intercept | | 5.9124 | | 3.9305 | | 1.50 | | 0.1461 | |
|  | Light | | 0.0013 | | 0.0006 | | 2.25 | | 0.0342 | |
|  | Photoperiod | | 0.9578 | | 0.2631 | | 3.64 | | 0.0014 | |
|  | Temperature | | -0.3552 | | 0.0704 | | -5.04 | | <.0001 | |
|  | Humidity | | -0.0991 | | 0.0192 | | -5.17 | | <.0001 | |
| Stepwise regression | | | |  | |  | |  | |  |
|  | ANOVA | | R^2^ | | R^2^ adj | | F4,23 | | P | |
|  |  | | 0.7081 | | 0.6573 | | 13.95 | | <.0001 | |
|  | Parameter Estimates | | | | | |  | |  | |
|  | Term | | Estimate | | Std Error | | t Ratio | | Prob>\|t\| | |
|  | Intercept | | 5.9124 | | 3.9305 | | 1.50 | | 0.1461 | |
|  | Light | | 0.0013 | | 0.0006 | | 2.25 | | 0.0342 | |
|  | Photoperiod | | 0.9578 | | 0.2631 | | 3.64 | | 0.0014 | |
|  | Temperature | | -0.3552 | | 0.0704 | | -5.04 | | <.0001 | |
|  | Humidity | | -0.0991 | | 0.0192 | | -5.17 | | <.0001 | |

1. **1^st^ – 2^nd^ instar larval development time**

| Regression | |  | |  | |  |  |
| --- | --- | --- | --- | --- | --- | --- | --- |
|  | ANOVA | | R^2^ | | R^2^ adj | F4,23 | P |
|  |  | | 0.68 | | 0.62 | 12.15 | <.0001 |
|  | Parameter Estimates | | | | |  |  |
|  | Term | | Estimate | | Std Error | t Ratio | Prob>\|t\| |
|  | Intercept | | 11.7509 | | 5.0614 | 2.32 | 0.0295 |
|  | Light | | 0.0013 | | 0.0008 | 1.73 | 0.0964 |
|  | Photoperiod | | 0.9648 | | 0.3388 | 2.85 | 0.0091 |
|  | Temperature | | -0.5290 | | 0.0907 | -5.83 | <.0001 |
|  | Humidity | | -0.1019 | | 0.0247 | -4.13 | 0.0004 |
| Stepwise regression | | | |  | |  |  |
|  | ANOVA | | R^2^ | | R^2^ adj | F3,24 | P |
|  |  | | 0.6367 | | 0.5913 | 14.02 | <.0001 |
|  | Parameter Estimates | | | | |  |  |
|  | Term | | Estimate | | Std Error | t Ratio | Prob>\|t\| |
|  | Intercept | | 16.8646 | | 4.2809 | 3.94 | 0.0006 |
|  | Photoperiod | | 0.7650 | | 0.3316 | 2.31 | 0.0300 |
|  | Temperature | | -0.5461 | | 0.0939 | -5.82 | <.0001 |
|  | Humidity | | -0.1114 | | 0.0250 | -4.45 | 0.0002 |

1. **3^rd^ – 4^th^ instar larval development time**

| Regression | |  | |  | |  | |  | |  |
| --- | --- | --- | --- | --- | --- | --- | --- | --- | --- | --- |
|  | ANOVA | | R^2^ | | R^2^ adj | | F4,23 | | P | |
|  |  | | 0.77 | | 0.73 | | 18.88 | | <.0001 | |
|  | Parameter Estimates | | | | | |  | |  | |
|  | Term | | Estimate | | Std Error | | t Ratio | | Prob>\|t\| | |
|  | Intercept | | 16.8227 | | 6.3257 | | 2.66 | | 0.0140 | |
|  | Light | | 0.0021 | | 0.0010 | | 2.18 | | 0.0401 | |
|  | Photoperiod | | 1.3505 | | 0.4234 | | 3.19 | | 0.0041 | |
|  | Temperature | | -0.8775 | | 0.1134 | | -7.74 | | <.0001 | |
|  | Humidity | | -0.0749 | | 0.0309 | | -2.43 | | 0.0233 | |
| Stepwise regression | | | |  | |  | |  | |  |
|  | ANOVA | | R^2^ | | R^2^ adj | | F4,23 | | P | |
|  |  | | 0.7666 | | 0.7260 | | 18.88 | | <.0001 | |
|  | Parameter Estimates | | | | | |  | |  | |
|  | Term | | Estimate | | Std Error | | t Ratio | | Prob>\|t\| | |
|  | Intercept | | 16.8227 | | 6.3257 | | 2.66 | | 0.0140 | |
|  | Light | | 0.0021 | | 0.0010 | | 2.18 | | 0.0401 | |
|  | Photoperiod | | 1.3505 | | 0.4234 | | 3.19 | | 0.0041 | |
|  | Temperature | | -0.8775 | | 0.1134 | | -7.74 | | <.0001 | |
|  | Humidity | | -0.0749 | | 0.0309 | | -2.43 | | 0.0233 | |

1. **Pupation time**

| Regression | |  | |  | |  |  |
| --- | --- | --- | --- | --- | --- | --- | --- |
|  | ANOVA | | R^2^ | | R^2^ adj | F4,23 | P |
|  |  | | 0.77 | | 0.73 | 19.46 | <.0001 |
|  | Parameter Estimates | | | | |  |  |
|  | Term | | Estimate | | Std Error | t Ratio | Prob>\|t\| |
|  | Intercept | | 22.5422 | | 6.9861 | 3.23 | 0.0037 |
|  | Light | | 0.0020 | | 0.0011 | 1.94 | 0.0643 |
|  | Photoperiod | | 1.3590 | | 0.4676 | 2.91 | 0.008 |
|  | Temperature | | -0.9833 | | 0.1252 | -7.85 | <.0001 |
|  | Humidity | | -0.0860 | | 0.0341 | -2.52 | 0.019 |
| Stepwise regression | | | |  | |  |  |
|  |  | |  | |  |  |  |
|  | ANOVA | | R^2^ | | R^2^ adj | F3,24 | P |
|  |  | | 0.7345 | | 0.7013 | 22.13 | <.0001 |
|  | Parameter Estimates | | | | |  |  |
|  | Term | | Estimate | | Std Error | t Ratio | Prob>\|t\| |
|  | Intercept | | 30.4556 | | 5.9958 | 5.08 | <.0001 |
|  | Photoperiod | | 1.0496 | | 0.4644 | 2.26 | 0.0332 |
|  | Temperature | | -1.0097 | | 0.1315 | -7.68 | <.0001 |
|  | Humidity | | -0.1007 | | 0.0351 | -2.87 | 0.0084 |

1. **Female emergence time**

| Regression | Regression | |  |  |  |
| --- | --- | --- | --- | --- | --- |
|  | ANOVA | R^2^ | R^2^ adj | F4,23 | P |
|  |  | 0.88 | 0.86 | 41.67 | <.0001 |
|  | Parameter Estimates | | |  |  |
|  | Term | Estimate | Std Error | t Ratio | Prob>\|t\| |
|  | Intercept | 29.8151 | 6.6810 | 4.46 | 0.0002 |
|  | Light | 0.0028 | 0.0010 | 2.77 | 0.0109 |
|  | Photoperiod | 1.4915 | 0.4472 | 3.34 | 0.0029 |
|  | Temperature | -1.3063 | 0.1197 | -10.91 | <.0001 |
|  | Humidity | -0.0609 | 0.0326 | -1.87 | 0.0744 |
| Stepwise regression | | |  |  |  |
|  | ANOVA | R^2^ | R^2^ adj | F3,24 | P |
|  |  | 0.8603 | 0.8429 | 49.28 | <.0001 |
|  | Parameter Estimates | | |  |  |
|  | Term | Estimate | Std Error | t Ratio | Prob>\|t\| |
|  | Intercept | 21.6131 | 5.2929 | 4.08 | 0.0004 |
|  | Light | 0.0032 | 0.0010 | 3.11 | 0.0047 |
|  | Photoperiod | 1.6361 | 0.4628 | 3.54 | 0.0017 |
|  | Temperature | -1.2365 | 0.1195 | -10.34 | <.0001 |

1. **Male emergence time**

| Regression | |  | |  | |  |  |
| --- | --- | --- | --- | --- | --- | --- | --- |
|  | ANOVA | | R^2^ | | R^2^ adj | F4,23 | P |
|  |  | | 0.79 | | 0.75 | 21.63 | <.0001 |
|  | Parameter Estimates | | | | |  |  |
|  | Term | | Estimate | | Std Error | t Ratio | Prob>\|t\| |
|  | Intercept | | 28.1664 | | 7.9326 | 3.55 | 0.0017 |
|  | Light | | 0.0023 | | 0.0012 | 1.97 | 0.0615 |
|  | Photoperiod | | 1.3760 | | 0.5310 | 2.59 | 0.0163 |
|  | Temperature | | -1.1468 | | 0.1422 | -8.07 | <.0001 |
|  | Humidity | | -0.0783 | | 0.0387 | -2.02 | 0.0547 |
| Stepwise regression | | | |  | |  |  |
|  | ANOVA | | R^2^ | | R^2^ adj | F2,25 | P |
|  |  | | 0.72 | | 0.69 | 31.61 | <.0001 |
|  | Parameter Estimates | | | | |  |  |
|  | Term | | Estimate | | Std Error | t Ratio | Prob>\|t\| |
|  | Intercept | | 46.1424 | | 5.3033 | 8.70 | <.0001 |
|  | Temperature | | -1.0095 | | 0.1283 | -7.87 | <.0001 |
|  | Humidity | | -0.1034 | | 0.0418 | -2.48 | 0.0205 |

1. **Adult daily survival rate**

| Regression | |  |  | |  | |  | |
| --- | --- | --- | --- | --- | --- | --- | --- | --- |
|  | ANOVA | R^2^ | R^2^ adj | F4,19 | | P | |  |
|  |  | 0.35 | 0.21 | 2.57 | | 0.071 | |  |
|  | Parameter Estimates | |  |  | |  | |  |
|  | Term | Estimate | Std Error | t Ratio | | Prob>\|t\| | |  |
|  | Intercept | 1.0185 | 0.0665 | 15.31 | | <.0001 | |  |
|  | light | 0.0071 | 0.0000 | 0.42 | | 0.6769 | |  |
|  | Photoperiod | 0.0377 | 0.0263 | 1.43 | | 0.1680 | |  |
|  | Humidity | -0.0032 | 0.0031 | -1.04 | | 0.3099 | |  |
|  | Temperature | -0.0157 | 0.0066 | -2.37 | | 0.0287 | |  |
| Stepwise regression | |  |  | |  | |  | |
|  | ANOVA | R^2^ | R^2^ adj | F1,22 | | P | |  |
|  |  | 0.23 | 0.20 | 6.61 | | 0.0174 | |  |
|  | Parameter Estimates | |  |  | |  | |  |
|  | Term | Estimate | Std Error | t Ratio | | Prob>\|t\| | |  |
|  | Intercept | 0.9940 | 0.0570 | 17.44 | | <.0001 | |  |
|  | Temperature | -0.0053 | 0.0020 | -2.57 | | 0.0174 | |  |

1. **Adult survival time**

| Regression | |  | |  | |  |  |
| --- | --- | --- | --- | --- | --- | --- | --- |
|  | ANOVA | | R^2^ | | R^2^ adj | F4,19 | P |
|  |  | | 0.50 | | 0.40 | 4.79 | 0.0076 |
|  | Parameter Estimates | | | | |  |  |
|  | Term | | Estimate | | Std Error | t Ratio | Prob>\|t\| |
|  | Intercept | | 13.3863 | | 3.5250 | 3.80 | 0.0012 |
|  | light | | -0.0005 | | 0.0009 | -0.53 | 0.5999 |
|  | Photoperiod | | 2.9011 | | 1.3955 | 2.08 | 0.0514 |
|  | Humidity | | -0.1366 | | 0.1629 | -0.84 | 0.4124 |
|  | Temperature | | -1.1317 | | 0.3510 | -3.22 | 0.0045 |
| Stepwise regression | | | |  | |  |  |
|  | No variable has been selected at P = 0.05 | | | | | | |

1. **Adult life-time egg mass produced**

| Regression | |  | |  | |  | |  | |
| --- | --- | --- | --- | --- | --- | --- | --- | --- | --- |
|  | ANOVA | | R2 | R2 adj | F4,19 | | P | |  |
|  |  | | 0.78 | 0.73 | 16.78 | | <.0001 | |  |
|  | Parameter Estimates | | | |  | |  | |  |
|  | Term | | Estimate | Std Error | t Ratio | | Prob>\|t\| | |  |
|  | Intercept | | -92.8739 | 39.3133 | -2.36 | | 0.0290 | |  |
|  | light | | -0.0370 | 0.0100 | -3.71 | | 0.0015 | |  |
|  | Photoperiod | | 2.8704 | 15.5638 | 0.18 | | 0.8556 | |  |
|  | Humidity | | 4.7812 | 1.8172 | 2.63 | | 0.0165 | |  |
|  | Temperature | | -6.2579 | 3.9148 | -1.60 | | 0.1264 | |  |
| Stepwise regression | | | |  | |  | |  | |
|  | ANOVA | | R2 | R2 adj | F3,20 | | P | |  |
|  |  | | 0.7789 | 0.7458 | 23.49 | | <.0001 | |  |
|  | Parameter Estimates | | | |  | |  | |  |
|  | Term | | Estimate | Std Error | t Ratio | | Prob>\|t\| | |  |
|  | Intercept | | -93.4750 | 38.2201 | -2.45 | | 0.0238 | |  |
|  | light | | -0.0385 | 0.0058 | -6.61 | | <.0001 | |  |
|  | Humidity | | 5.0871 | 0.7243 | 7.02 | | <.0001 | |  |
|  | Temperature | | -5.6137 | 1.7245 | -3.26 | | 0.004 | |  |
