# Supplementary material for: Semi-field life-table studies of Aedes albopictus (Diptera: Culicidae) in Guangzhou, China
Source: PLoS One. 2020 Mar 18;15(3):e0229829. doi: 10.1371/journal.pone.0229829 (PMC7080243; doi:10.1371/journal.pone.0229829)
Supplement: Attachment — Submitted filename: Reviewer reports.docx2019.12.7.docx [file pone.0229829.s007.docx]

**Reviewer reports:**

**Reviewer #1:** The manuscript should be corrected by a professional English person. After the correction the MS can accepted for the publication in this journal.

**Response:** Thank you for your comments and suggestions. The language in the revised manuscript has been polished by a native English editor.

**Reviewer #2:** Overall, I think this study contains useful and publishable data. The authors track larval and adult development and survival of *Aedes albopictus* in field conditions. There are several elements of the experimental design and analysis that need to be made clearer. Data is collected during two time periods, Sept-Jan and June-July. From what I can tell there are 4 time points in the Sept and Jan period and 2 in June July. From, Fig 4 it appears these run once a month with an unexplained gap in the data between Jan-May. Further, from lines 148-155 it looks like different generations were used in different experiments. In the Sept-Jan data experiments were conducted in the "semi-field" setting and then experiments move to a "semi-natural" setting in June-July. It wasn't clear to me why this move occured or how differences between these setting might influences the conclusions of the study. It was also unclear to me from how the results are currently presented how much this data can tell us generally about the effects of temperature, RH, and light intensity on these different mosquito traits. There is a comparison of months. Instead of this type of comparison, temperature, humidity and other environmental variables could be used in models to determine their relative impacts on the different measures of mosquito development and survival. I do think the study clearly provides insight into whether *Ae. albopictus* could sustain populations in early winter in Guangzhou.

Overall, I feel that if the methods are made much clearer and the results are presented in terms of the strength of the environmental variables recorded as predictors this would be a nice ms.

**Response:** We thank the reviewer’s comments and suggestions. We have revised those points made by the reviewer. We did univariate and multiple regression analysis and added a summary table (Table 3) to summarize the correlations between larval development, adult survivorship and environmental variables. We have also added one section to describe these new results. We hope this makes the results much clearer. The change from one place to another place was because the place we used for the September-November experiments was not available anymore. The environment of the two settings are similar, all are in semi-open area with near-natural temperature and humidity and half-shed lights. We described this in the revised manuscript.

**The reviewer’s questions:**

(1) There are several elements of the experimental design and analysis that need to be made clearer. Data is collected during two time periods, Sept-Jan and June-July. From what I can tell there are 4 time points in the Sept and Jan period and 2 in June July. From, Fig 4 it appears these run once a month with an unexplained gap in the data between Jan-May.

**Response:** Yes, the reviewer was correct, we have done larval and adult life-tables 4 times from August to November (adult life-table continued through January next year) and 2 times in June and July. We stated so in the Methods sections.

(2) It was also unclear to me from how the results are currently presented how much this data can tell us generally about the effects of temperature, RH, and light intensity on these different mosquito traits. There is a comparison of months. Instead of this type of comparison, temperature, humidity and other environmental variables could be used in models to determine their relative impacts on the different measures of mosquito development and survival.

**Response:** We did univariate and multiple regression analysis, the results are shown in Table 3 (new table) and we added one section to describe these new results.

**Reviewer #3:** This study describe major finding on *Aedes albopictus* larvae could develop and emerge, and adults could survive and produce eggs in early winter in Guangzhou under semi-field conditions. It is of interest and could reflect climate change and explain dengue cases caused by *Ae. albopictus* appeared in Henan province in temperate central China. However, I suggest authors to provide more information on the differences between laboratory conditions and semi-field conditions when using the same mosquito sources from field.

**Response:** Thank you for your comments and suggestions.

**The reviewer’s questions:**

Introduction

1. Although the temperature and humidity are key factors for mosquito growth, pathogen of virus and plasmodium are quite different, and may not be suitable to compare together. Besides, behavior of diurnal and nocturnal are also reflect on their adaptation on environmental conditions. Please re-construct your sentences and remove the malaria reference (Line81-86; Line 98-101) if you intent to focus on *Ae. albopictus* for dengue in China.

**Response**：On the basis of your suggestions, we have revised the sentences, removed the malaria reference in lines 81–86, and rewritten this section.

**Materials and Methods Questions:**

(1) According to authors’ description:… (Line126) winter temperatures can drop below 10°C, and annual rainfall is about 1,678 mm (Figure. 2). This climate is ideal for the development and reproduction of *Ae. albopictus* (Li et al. 2014).…. It means mosquitoes can hide in some place to survive or delayed in development. Can authors provide more information or reference that study mosquito life cycle in low temperature?

**Response：**We have rewritten this sentence and cited reference [41]. (revised version: lines 114-115).

(2) Line 133: Field collected larvae from different habitats were mixed by putting larvae from different habitats into the same bucket….I am curious is it happened to find mosquito larvae during winter?

**Response**：This is a misunderstood, we described that “field strain *Ae. albopictus* larvae collected in May 2017 from multiple (>10) breeding habitats in two residential areas …,” no other mosquito larvae have been collected in this study afterward.

In other studies, researchers did find *Aedes albopictus* larvae in winter in Guangzhou, and the larvae could stay over winter (Zhong Xue-shan, XIAO Xiao-ling, XIANG Ying-fei. Overwintering breeding site types and larvae survival of Aedes albopictus in Yuexiu District, Guangzhou. South China J Prev Med. 2019; 45(4): 386-388,397).

(3) Line 141; rodent blood or mouse blood?

**Response:** mouse blood.

(3) Line 143: please describe food source of mosquito adult in Semi-field setting and Semi-natural setting.

**Response：**We have described the food source in lines 184–186 (revised version: line 172). “The mosquitoes were provided with 10% glucose daily, and, every three days, a mouse was placed in each cage for approximately 4 h to blood-feed the mosquitoes.”

(5) Line 192: Please explain the study design.

**Response：**The study design has been described in four sections.

(6) Line 194: sex ratio of adult mosquitoes?

**Response**：We added sex ratio in the results.

**Results questions**

1. Line 213: Why record humidity in larvae experiments?

**Response:** Humidity and temperature are the two key factors affecting mosquito development, it is a ‘must be observed’ measurement during life-table studies.

(3) Line 223: Octerber should be revised as October.

**Response**：We have made the required revision.

(3) Line 234, Line 410: Please provide the method of egg hatch in materials and methods part. And how you count the number of larvae or reared-egg.

**Response:** We collected the eggs with a moist filter paper. The fresh eggs were fully developed in a humidified anoxic environment for 2 days and then manually counted. The larvae were counted manually every day, We described these in the revised manuscript.

Discussion questions

(1) Line 308: stress.The => stress. The

**Response：**We have made the required modification.

⑵Discussion should be shorted. Line 375: discussion of vectorial capacity need to be removed.

**Response：**The discussion of vectorial capacity has been removed from the manuscript.

(3)	Line 386: 2015,Maimusa et al. => 2015, Maimusa et al.

**Response：**We have made the required correction.

(4) Line 420: it’s Anopheles mosquitoes, about malaria, not Aedes mosquitoes.

**Response：**We completely agree with the reviewer; we have revised the sentences and removed the malaria reference.

(5) Line 432: Ae. aegypti => Ae. Aegypti

**Response：**We have made the required correction.

(6)Line 442: Afrane et al. and Imam et al. => Afrane et al. and Imam et al.

**Response：**We have made the required correction.

(7)Line 445: Villarreal wt al. 2018 => Villarreal et al. 2018

**Response：**We have made the required correction.

(8) Line 458: Lastly but not the least, in this study, we didn’t check blood meal status of experimental adults after blood feeding,……this is very important, also do you try to try to find dead male mosquito to ensure that mosquitoes has finished their mating.

**Response：**In the experiment, we recorded the number of dead males and females every day and removed the dead individuals.

**Reviewer #4:** General comments

This manuscript describes the impact of semi-field conditions on life-table parameters of Ae. albopictus. This study for most parts is well designed, executed presented. However, the manuscript falls short in the way the data has been analysed and the results have been visualized.

**Response：**Thank you for your comments and suggestions.

**Major points**

1. The authors claim to have employed life-table experiments to identify the lowest temperature that supports the survival and reproduction of *Ae. albopictus*. In this study, larval development and survival, and adult survival have been quantified as dependent variables. Further, lifetime egg mass is mentioned as a proxy for adult reproduction but there is neither a mention of how it was quantified nor how well it correlates with lifetime fecundity of mosquitoes. All these quantified variables are life-history traits in mosquitoes and cannot be considered as life-table parameters. This study does not use these quantified life-history traits to estimate life-table parameters such as net reproductive rate or the cohort rate of increase. A typical example describing how life-table analysis could be estimated from life-history traits can be found here: <https://doi.org/10.1371/journal.pone.0180093>

**Response:** We fully agree with the reviewer, life-history traits can be estimated from life-table analysis. The purpose of this study is to determine how environmental factors such as temperature, humidity and photoperiod affect larval development and adult survivorship, the key focus is “can *Aedes albopictus* survive and produce viable eggs in winter in Guangzhou?” This is important for the assessment of risks of dengue and other mosquito-borne diseases. Accurate quantification of life history traits is not the focus of the study.

2. The authors have performed experiments in semi-field and semi-natural settings. The purpose of these two treatments is not justified. How do these two treatments address the objective of this study?

**Response:** Most life-table studies are carried out under laboratory conditions, results may not be reflective of actual development in fluctuated natural conditions. Since fully natural experiments are ethically not allowed, semi-natural and semi-field condition experiments are the best possible approximation.

3. Male and female mosquitoes develop at distinct rates (protandry) and are sexually size dimorphic. As a consequence, the effects of larval growing conditions on adult mosquitoes differentially impact males and females during the adult stages. Therefore, to assess the effects of temperature on mosquito life-history traits accurately, data in this study (figures 5-7, 8a, 8b, and 9) will have to be analyzed separately for male and female mosquitoes.

**Response:** We fully agree with the reviewer, we have done to analyses separately for male and female mosquitoes.

4. Adding to my earlier comment on lifetime egg mass estimation (pt.1), it is unclear if the methods employed by the authors were good enough to estimate the lifetime fecundity or just fecundity in the first gonotrophic cycle. The authors will have to clarify how this method is best suited to estimating life-table traits.

**Response:** We actually collected all eggs not just in the first gonotrophic cycle. The small number of average lifetime egg mass was likely due to the short adult survival time, most females dead before eggs were first collected. We have discussed this in the discussion section.

5. Based on adult survivorship curves, the authors have concluded that percentage adult survival could have implications on mosquito-borne disease transmission. While this is partly true, recent studies have identified that only 20% of mosquitoes in a population contribute to 80% of the disease transmission [1,2]. It will be worthwhile to discuss the results of this study in light of these earlier findings. Also, no hazard ratios were reported from the Kaplan-Meier survival analysis.

**Response:** We fully agree with the reviewer, we have discussed this in the discussion section and added three references [49-51].

[49] Liu Z, Zhou T, Lai Z, Zhang Z, Jia Z, Zhou G, Williams T, Xu J, Gu J, Zhou X, Lin L, Yan G, Chen XG. Competence of Aedes aegypti, Ae. albopictus, and Culex quinquefasciatus mosquitoes as Zika virus vectors, China. Emerg Infect Dis. 2017;23(7):1085-1091.

[50] Mariconti M, Obadia T, Mousson L, Malacrida A, Gasperi G, Failloux AB, Yen PS. Estimating the risk of arbovirus transmission in Southern Europe using vector competence data. Sci Rep. 2019;9(1):17852.

[51] Churcher TS, Trape J-F, Cohuet A. Human-to-mosquito transmission efficiency increases as malaria is controlled. Nat Commun. 2015; 6: 6054.

6. How was the larval density (group size) and nutrition (0.1g turtle food per 100 larvae) determined? What is the rationale behind choosing these values for this study?

**Response:** The amount of food supplied was based on observations. If larval food has a lot leftovers from previous day, which means the food supply was maybe too much, therefore no additional food should be added; on the other hand, if larval food was consumed earlier, additional food should be supplied. The amount of 0.1g per 100 larvae was an estimation not an accurate amount, because food was added more often for old larvae compare to young larvae.

7. This study does not discuss the links between the results of this study and the risk of dengue transmission. More information on how these findings could be of use in vector control approaches/strategies or predictive modelling of disease outbreaks is desirable.

**Response:** We agree with the reviewer, we have discussed these points.

**Minor points**

1. Do Ae. albopictus diapause in the study area? If yes, at what temperature?

**Response:** We did not confirm any diapause during the study. But possibly observed the larval diapause at a daily average temperature of 10.9°C (ranged from 2.1 – 21.2°C). Possible diapause was observed in 4th instar larvae during the winter experiment, however not confirmed. Therefore, diapause was not reported.

2. Lines 456-457: The claim need not be true because the strains of mosquitoes and several other experiment factors could vary between studies. Therefore, the results might not be comparable across studies.

**Response:** Environmental conditions in Semi-field and Semi-natural settings are very similar, we changed the location because of the availability of the facilities.

3. Line 251: Using the term ‘immature’ to refer to larvae is fine. But here it has not been done on a consistent basis.

Response: We agree with the reviewer and have made the required revisions.
