# Supplementary material for: Semi-field life-table studies of Aedes albopictus (Diptera: Culicidae) in Guangzhou, China
Source: PLoS One. 2020 Mar 18;15(3):e0229829. doi: 10.1371/journal.pone.0229829 (PMC7080243; doi:10.1371/journal.pone.0229829)
Supplement: Attachment — Submitted filename: Reviewer reports.docx2020.1.12.docx [file pone.0229829.s008.docx]

**Reviewer reports:**

Reviewer #2: Thank you for your responses. I have just one more set up suggestions for reporting your environmental analyses. These are really nice and I think have greatly improved the manuscript.

**Response:** We thank the reviewer’s constructive comments and suggestion.

1. Since your data on humidity, temperature, and length of photoperiod were collected as part of one study, I would recommend just using your multivariate analyses because the univariate analyses would be driven by correlations between your environmental variables.

**Response:** Multiple regression analyses (both general and stepwise) have been done on all life table outcomes, Table 3 has been updated, a supplementary table was added to show all the regression results.

2. Instead of only reporting significance of correlations can you describe the relationship biologically? For example instead of " In the pupation rate analysis, temperature was the only significant variable (P = 0.0164) that positively affected pupation rate" you could say something like "Pupation rate increased with environmental temperature (P=0.0164)" or "Females developed faster in warmer conditions and more slowly when humid was lower."

**Response:** Similar statements have been rewritten to describe the biological relationship.

Reviewer #3: This study describe major finding on Aedes albopictus larvae could develop and emerge, and adults could survive and produce eggs in early winter in Guangzhou under semi-field conditions. It is of interest and could reflect climate change and explain dengue cases caused by Ae. albopictus appeared in Henan province in temperate central China. This manuscript has been revised accordingly and suggested to be published on this journal.

**Response:** We thank the reviewer’s constructive comments.

Reviewer #4: The authors have responded to all the queries raised by the reviewers. However, one of my points still remains unaddressed: The authors claim that this is a semi-field life-table study. However, this study does not quantify any of the life-table traits. This study quantifies select larval and adult traits that are known to correlate with life-table characteristics of mosquitoes. Since life-table analyses are missing in this study, the authors will have to remove the term 'life-table' from this manuscript. They could rather claim that they have employed mosquito traits as a proxy to understanding life-table traits in mosquitoes using a semi--field study.

**Response:** We agree with the reviewer, we re-stated that this study “employed mosquito traits as a proxy to understanding life-table traits in mosquitoes using a semi – field study” in the “Methods” section and in “Abstract” as the reviewer suggested.
